# *BRCA1* promoter hypermethylation is not associated with germline variants in Polish breast cancer patients

**DOI:** 10.1186/s13053-025-00317-8

**Published:** 2025-06-10

**Authors:** Karolina Prajzendanc, Paweł Domagała, Jolanta Hybiak, Wojciech Kluźniak, Cezary Cybulski, Katarzyna Białkowska, Alicja Ogrodniczak, Janusz Ryś, Aleksandra Sejda, Marek Szwiec, Joanna Tomiczek-Szwiec, Tomasz Kluz, Roksana Dwornik, Dagmara Cylwik, Jacek Gronwald, Jan Lubiński, Anna Jakubowska

**Affiliations:** 1 Department of Genetics and Pathology, International Hereditary Cancer Center, Pomeranian Medical University, Szczecin, Poland; 2 Department of Clinical Genetics and Pathology, University of Zielona Góra, Zielona Góra, Poland; 3 Department of Pathology, Pomeranian Medical University, Szczecin, Poland; 4 Department of Tumor Pathology, Maria Skłodowska-Curie Memorial Centre and Institute of Oncology, Cracow, Poland; 5 Department of Pathomorphology and Forensic Medicine, University of Warmia and Mazury, Olsztyn, Poland; 6 Department of Surgery and Oncology, University of Zielona Góra, Zielona Góra, Poland; 7 Department of Clinical Oncology, University Hospital in Zielona Góra, Zielona Góra, Poland; 8 Clinical Department of Oncological Gynecology at the Oncology Centre in Opole, University of Opole, Opole, Poland; 9 Department of Biology and Genetics, Faculty of Medicine, University of Opole, Opole, Poland; 10 Institute of Obstetric and Emergency Medicine, Faculty of Medicine, University of Rzeszow, Rzeszow, Poland; 11 Independent Laboratory of Molecular Biology and Genetic Diagnostics, Pomeranian Medical University, Szczecin, Poland

**Keywords:** *BRCA1* gene, Methylation, Breast cancer

## Abstract

**Background:**

Methylation of *BRCA1* has been associated with an increased risk of breast cancer and specific clinical characteristics of the disease. In the British population, the genetic alteration c.-107 A/T has been shown to cause allelic methylation, leading to familial breast and ovarian cancer. However, this variant has not been detected in Polish population. Nonetheless, other genetic variants may still be associated with *BRCA1* methylation, highlighting the need for further research.

**Aim of study:**

This study aimed to analyze *BRCA1* promoter region to identify germline alterations associated with *BRCA1* methylation in peripheral blood DNA. Additionally, the correlation between the detected variants and breast cancer incidence, as well as clinical characteristics, was assessed.

**Materials and methods:**

One hundred breast cancer patients with *BRCA1* methylation were analyzed using pyrosequencing to quantify methylation levels. Immunohistochemistry (IHC) was performed on formalin-fixed paraffin-embedded (FFPE) tumor tissue samples from these patients to assess BRCA1 expression. In 47 patients with the highest *BRCA1* methylation and decreased BRCA1 expression, Sanger sequencing was performed encompassing 643 bp of *BRCA1* promoter to identify potential variants associated with methylation and/or breast cancer. A variant was identified and genotyped in multiple patient groups, including 336 *BRCA1* methylation-positive women, 1898 unselected breast cancer cases, 2234 healthy controls, and 309 *BRCA1* pathogenic variant (PV) carriers. Statistical analyses were performed using Fisher’s exact test and Chi-square test in Stata/IC version 16.1.

**Results:**

The study identified *BRCA1* c.20 + 101 C/G (rs799905) in the promoter region of the gene. However, no significant association was found between rs799905 and *BRCA1* methylation or breast cancer risk. Nevertheless, a borderline association was observed between rs799905 and the occurrence of triple-negative breast cancer (TNBC) (*p* = 0.048) as well as lymph node (LN) metastases (*p* = 0.046). Among *BRCA1* PV carriers, the GG genotype was significantly more frequent, while the CC genotype was 10 times less common compared to non-carriers (*p* = 0.003).

**Conclusion:**

Rs799905 variant is not associated with *BRCA1* methylation breast cancer risk. However, it may be linked to the occurrence of TNBC and LN metastases. Additionally, rs799905 may be associated to some extent with *BRCA1* PVs, though further research is needed to clarify the nature of this potential correlation.

## Background

DNA methylation is an epigenetic mechanism that targets cytosines adjacent to guanines (CpG dinucleotides), which are often found in CpG islands within gene promoters and repetitive DNA regions [1]. The overall methylation pattern (methylome) is established after fertilization and maintained throughout development [2]. In healthy cells, repetitive DNA regions are methylated to preserve genomic stability, whereas gene promoters typically remain unmethylated to enable gene expression [1]. This process is essential for cell differentiation and tissue specificity, leading to tissue-specific methylation patterns based on functional requirements [3]. DNA methylation has been implicated in carcinogenesis [1]. Somatic inactivation of tumor suppressor genes, such as *BRCA1* and *MLH1*, through promoter methylation leads to gene silencing and contributes to cancer development [4, 5]. Additionally, promoter methylation detected in peripheral blood DNA has been associated with an increased cancer risk, similar to germline PVs [6]. It has been suggested that aberrant promoter methylation may result from germline DNA sequence variants acting in cis [7]. These genetic alterations could help explain the missing heritable component of cancer in patients who lack detectable germline PVs in gene’s coding sequence [8]. This phenomenon has been described for *MLH1* [9, 10] and *MSH2* [11], where specific germline variants in 5’UTR were linked to promoter methylation, leading to loss of expression and causing hereditary colorectal cancer. A cis-acting germline alteration located in the 5’ UTR of *BRCA1* gene (c.-107A > T, rs2154580237), has been previously identified in 2018 in two British families and was found to segregate with *BRCA1* promoter hypermethylation, predisposing carriers to breast and ovarian cancer [12]. However, this variant was not detected in 193 Dutch [13] and 3297 German [14] patients. It was also not found in 942 Polish breast cancer patients and 500 controls [unpublished data]. Nonetheless, it remains possible that other germline variants in the *BRCA1* 5’ UTR region are associated with promoter hypermethylation and may be connected with cancer risk in different populations.

In this study, we aimed to analyze the *BRCA1* promoter region to identify germline alterations associated with *BRCA1* methylation in peripheral blood DNA. Additionally, detected variants were assessed and correlated with breast cancer incidence and other clinical characteristics.

## Materials and methods

The study was conducted in 2 related analyses:

1. Identification of germline alterations in 5’UTR/promoter region of *BRCA1* gene associated with methylation detected in peripheral blood DNA.
2. Association analysis and clinical characteristics of identified germline variants in Polish breast cancer patients.

### Study population

1. The initial study group consisted of 100 breast cancer (BC) patients with previously detected *BRCA1* promoter methylation in peripheral blood and paired tumor tissue DNA with the MS-HRM technique [15]. Among them 47 BC patients selected on the results of pyrosequencing and BRCA1 expression in tumor tissue that subsequently were analyzed by Sanger sequencing for germline alterations associated with *BRCA1* promoter methylation in peripheral blood DNA. All patients were negative for the common Polish germline *BRCA1/2* PVs [16, 17].
2. Association analysis of germline variant identified by sequencing was performed in 3 independent groups:
  Group I: 336 women with detected *BRCA1* promoter methylation in peripheral blood, consisted of 189 BC patients and 147 healthy controls [15]; 109 healthy women were from an ongoing prospective study.
  Group II: 1898 unselected BC patients and 2234 healthy controls.
  Group III: 309 BRCA1 PV carriers consisting of 41 BC patients and 268 healthy women.

In Group II and Group III status of *BRCA1* promoter methylation was unknown.

In breast cancer patients from Group II clinical and histopathological data including age of diagnosis, age of death, cancer type, hormone receptor status, tumor size, metastases, bilaterality, family history and *BRCA1* PV status were collected.

All individuals for this study were selected from the registry of International Hereditary Cancer Center, Department of Genetics and Pathology Pomeranian Medical University in Szczecin. Each patient has signed an informed consent for study participation.

The study was approved by Ethics Committee of the Pomeranian Medical University in Szczecin.

### Genomic DNA isolation

The DNA isolation was performed using the detergent method as previously described [18]. After isolation the DNA samples were stored at 4°C prior to analysis.

### Pyrosequencing of *BRCA1* promoter region

Quantitative DNA methylation analysis by pyrosequencing was performed on peripheral blood DNA samples of 100 breast cancer patients (with detected *BRCA1* methylation, as indicated in the Study population), which was previously bisulfite converted with the use of EZ DNA Methylation Kit (Zymo Research) according to the manufacturer’s protocol. Pyrosequencing was performed on the PyroMark MD Instrument (Biotage) according to manufacturer’s procedure. The 6 CpG sites in *BRCA1* promoter region were examined (+ 8, + 14, +16, + 19, +27, + 44). Primer sequences used for pyrosequencing are presented in Table 1. The methylation levels of the individual CpG sites varied between 4% and 25% across all samples. The average methylation level of the samples ranged from 5 to 21%.

**Table 1:** Sequences of primers used in pyrosequencing (5’→3’)

Primer F	GGGTAGATTGGGTGGTTAATT
Primer R^a^	TTATCTAAAAAACCCCACAACCTATC
Primer S	GGGAATTATAGATAAATTAAAATTG

^a^biotinylated

### BRCA1 expression

Expression of BRCA1 was performed by immunohistochemistry (IHC) and encompassed 100 BC tissue samples (with previously detected *BRCA1* gene methylation, as indicated in Study population). Tissue microarrays of BC patients were constructed as described previously [19]. The slides were immunostained using a Dako EnVision FLEX + visualization system according to the manufacturer’s instructions. Mouse monoclonal BRCA1 antibody (clone MS110, Calbiochem; dilution 1: 50; incubation time 30 min) was used. The expression of BRCA1 protein was classified as negative (< 10% positive tumor nuclei) or positive (≥ 10% tumor cells showing nuclear immunoreactivity).

### Sanger sequencing of *BRCA1* 5'UTR

Sequencing of 5’UTR region of *BRCA1* gene, encompassing 643 nucleotides, has been performed on samples initially analyzed by pyrosequencing (quantitative DNA methylation analysis) and immunohistochemical analysis of tissue microarrays (BRCA1 expression). Out of the 100 patients tested, 47 were selected for Sanger sequencing. These patients exhibited the highest average levels of *BRCA1* methylation (> 14%) and negative BRCA1 protein expression (< 10% of cells showing immunoreactivity) in paired tumor tissues. The sequencing was performed using the BigDye Terminator v3.1 Cycle Sequencing kit (Life Technologies, Carlsbad, California) according to commonly used protocols. The sequencing products were analyzed using the ABI Prism 3500XL Genetic Analyzer (Life Technologies). Sequences of primers are presented in Table 2.

**Table 2:** Sequences of primers used in Sanger sequencing (5’→3’)

Primer F	ACTGTGATGCAATAAGCCGC
Primer R	GCATATTCCAGTTCCTATCACG

### Genotyping

Germline variant identified by sequencing has been genotyped in 3 study groups by Real-time PCR using TaqMan probes on LightCycler 480 Instrument (Roche Diagnostics) according to standard protocol. The reaction mix for each sample consisted of GoTaq^®^ Probe qPCR Master Mix (Promega), TaqMan Genotyping Assay × 40 (Predisigned, C_3178681_10; Applied Biosystems), and deionized water (Promega). Samples were analyzed on 384well plates. Each plate included a negative control with water and positive controls for each genotype.

### Statistical analysis

The frequency of each genotype for the detected variant was calculated. Then, for each genotype, odds ratios (OR) with 95% confidence intervals (CI) were determined by comparing it to the reference (most frequent) genotype using Fisher’s exact test. The correlation of detected variants with clinico-pathological features of breast cancers has been analysed using the Chi-square test for the categorical variables. *P*-values < 0.05 were considered statistically significant. All statistical analyses have been performed with the use of Stata/IC version 16.1.

## Results

### Identification of germline variant in promoter region of *BRCA1* gene

Sequencing of the promoter region revealed the presence of a single variant in *BRCA1* c.20 + 101 C/G (rs799905), localized in first intron between alternative exons 1α and 1β. Among 47 women rs799905 was detected in 18 patients (38.3%), including 3 homozygotes.

### Association analyses of *BRCA1* c.-20 + 101 C/G (rs799905)

The genotype frequency of rs799905 variant was similar in Groups I and II, with the genotype GG being the most common in BC patients (44.4% and 46%) and controls (46.9% and 44.5%), followed closely by genotype GC (BC cases 41.3% and 40.5%, controls 40.1% and 43.8%); the genotype CC was detected in 13.2% and 10.4% of BC patients, and 8.8% and 10% controls from Group I and II, respectively. In contrast, the frequencies of *BRCA1* c.-20 + 101 C/G in Group III (consisted of *BRCA1* PV carriers) differed significantly from those in Groups I and II. The most common GG genotype was detected in 69.5% BC patients and 61.9% healthy controls. Genotype GC was less frequent, appearing in 29% of cases and 37% of controls. The largest difference between the tested groups was observed in the frequency of genotype CC, which was detected in only 1.2% BC cases and 0.9% of controls in Group III. Association analyses of rs799905 did not show any statistically significant correlation with breast cancer in the 3 studied groups. Genotyping results and calculated odds ratios are presented in Table 3.

**Table 3:** Association of rs799905 with breast cancer in 3 tested groups

Group I	**Genotype**	**Cases Met (+) ** ***n*** ** = 189 (%)** ^a^	**Controls Met (+) ** ***n*** ** = 147 (%)** ^a^	**OR (95%CI)**	***p*** **-value**
	GG	84 (44.4)	69 (46.9)	Ref.	-
	GC	78 (41.3)	59 (40.1)	1.09 (0.66–1.78)	0.81
	CC	25 (13.2)	13 (8.8)	1.58 (0.71–3.62)	0.27
Group II	**Genotype**	**Cases**,***n* = 1898 (%)**^b^	**Controls**,***n* = 2234 (%)**^b^	**OR (95%CI)**	***p*** **-value**
	GG	874 (46)	995 (44.5)	Ref.	-
	GC	768 (40.5)	978 (43.8)	0.89 (0.78–1.02)	0.09
	CC	198 (10.4)	223 (10)	1.01 (0.81–1.26)	0.96
Group III	**Genotype**	**Cases**,***n* = 82 (%)**^c^	**Controls**,***n* = 268 (%)**^c^	**OR (95%CI)**	***p*** **-value**
	GG	57 (69.5)	166 (61.9)	Ref.	-
	GC	24 (29.3)	100 (37.3)	0.70 (0.39–1.23)	0.23
	CC	1 (1.2)	2 (0.7)	1.46 (0.02–28.4)	1.00

^a^2 (1%) cases and 6 (4%) controls failed to genotype

^b^58 (3,1%) cases and 38 (1,7%) controls failed to genotype

^c^comprise 41 patients from unselected breast cancer cases

### Association of *BRCA1* c.-20 + 101 C/G (rs799905) with clinical characteristics

The clinical characteristics were analyzed among unselected breast cancer cases from Group II. In this analysis the correlation of rs799905 genotypes with clinical and pathological features of breast cancers was assessed (Table 4). Results showed a higher frequency of GG than GC or CC genotypes (16.1% vs. 11.3% or 9.6% respectively) in patients with TNBC (triple negative BC), and CC than CG or GG genotypes (54.7% vs. 44.7% or 43.1% respectively) in patients with lymph node (LN) metastases. However, these observations were borderline statistically significant (*p* = 0.48 for TNBC and *p* = 0.46 for LN metastases). There was also a statistically significant association of rs799905 with presence of germline *BRCA1* PV (*p* = 0.003). All correlations of rs799905 with BC characteristics are presented in Table 4.

**Table 4:** Association of rs799905 with breast cancer characteristics in group of unselected cases

**Characteristic**	**Genotype**			***p*-value**
	**GG **	**GC **	**CC **	
Age of onset, years				0.98
Mean	54	53.7	53.8	
Median	52	52	53	
Age of onset below 50, n (%)				0.62
Yes	395 (45.2)	344 (44.8)	82 (41.4)	
No	479 (54.8)	424 (55.2)	116 (58.6)	
Death, n (%)				0.48
Yes	146 (16.8)	111 (14.6)	30 (15.5)	
No	723 (83.2)	648 (85.4)	163 (84.5)	
No data: 19				
Histopathological type, n (%)				0.54
Ductal G1/G2	276 (40.6)	242 (40.9)	72 (43.9)	
Ductal G3	135 (19.9)	100 (16.9)	25 (15.2)	
Ductal Gx	57 (8.4)	50 (8.4)	11 (6.7)	
Lobular	87 (12.8)	90 (15.2)	31 (18.9)	
Medullar	19 (2.8)	18 (3)	5 (3)	
Tubular	10 (1.5)	8 (1.3)	1 (0.6)	
DCIS	27 (4)	18 (3)	1 (0.6)	
Other	69 (10.1)	66 (11.1)	18 (11)	
No data: 404				
TNBC, n (%)				**0.048**
Yes	79 (16.1)	47 (11.3)	11 (9.6)	
No	411 (83.9)	369 (88.7)	103 (90.4)	
No data: 820				
Tumor size, n (%)				0.71
<1cm	71 (12.4)	66 (13.2)	15 (11.4)	
1-1.9	225 (39.3)	210 (42.1)	52 (39.4)	
2-4.9	250 (43.6)	208 (41.7)	61 (46.2)	
>5	27 (4.7)	15 (3)	4 (3)	
No data: 636				
Lymph nodes metastases, n (%)				**0.046**
Yes	248 (43.1)	231 (44.7)	76 (54.7)	
No	328 (56.9)	286 (55.3)	63 (45.3)	
No data: 608				
Bilateral BC, n (%)				0.47
Yes	34 (5.2)	22 (3.8)	6 (4.1)	
No	615 (94.8)	556 (96.2)	141 (95.1)	
No data: 466				
BC/OC among relatives, n (%)				0.24
Yes	36 (4.1)	21 (2.7)	7 (3.5)	
No	838 (95.9)	747 (97.3)	191 (96.5)	
Germline* BRCA1* PV, n (%)				**0.003**
Yes	30 (3.4)	10 (1.3)	1 (0.5)	
No	844 (96.6)	758 (98.7)	197 (99.5)	

*DCIS* Ductal carcinoma in situ, *TNBC* Triple-negative breast cancer, *BC* Breast cancer, *OC* Ovarian cancer, *PV* Pathogenic variant

## Discussion

Although DNA methylation has been investigated for many years, its precise molecular mechanism remain unclear. Various factors, including genetic predisposition and random environmental influence, appear to contribute to the methylation of multiple genes across different tissues and at various stages of life. This study aimed to explore and analyze the genetic basis of *BRCA1* promoter methylation in peripheral blood DNA and its potential contribution to breast carcinogenesis.

It has been suggested that gene methylation resulting from germline alterations may contribute to a subset of inherited cancers in families without a detectable predisposing PV [7, 8]. This theory assumes that higher (~ 50%) level of (constitutional) DNA methylation detected in patients would indicate the modification of a single allele, likely caused by cis-acting germline variant localized in promoter region of the gene.

For our study, we selected group of 100 breast cancer patients who had previously been analyzed by MS-HRM and showed *BRCA1* promoter methylation in peripheral blood and paired tumor tissue DNA [15]. In this group, we investigated DNA methylation using quantitative pyrosequencing to identify cases with possible allelic methylation of *BRCA1* promoter. However, in our study group of 100 breast cancer cases, the detected methylation levels ranged from 4 to 25% and were lower than the expected ~ 50% (corresponding to allelic methylation of *BRCA1* gene). Nonetheless, based on the combined results from pyrosequencing (quantitative methylation) and immunohistochemistry (BRCA1 protein expression), we sought to identify germline alterations that might be associated with *BRCA1* promoter methylation and correlate with breast cancer risk. In 47 out of 100 tested patients with highest methylation level (> 14%) and absent or low BRCA1 expression in tumor tissue (< 10% cells with immunoreactivity), we searched for germline alterations in the *BRCA1* promoter region. Sequencing of 643 bp fragment encompassing promoter of *BRCA1* gene identified a single intronic variant in *BRCA1* c.-20 + 101 C > G, located between alternative exons 1α and 1β of the gene. This variant was reported in NCBI databases (rs799905) and assumed to be clinically benign, primarily based on the minor allele frequency (MAF) of 28% in the European population.

In our study, rs799905 was detected in 18 out of 47 (38%) BC patients with detectable methylation and low expression of BRCA1; subsequently it was tested in 3 study groups to evaluate a possible association with breast cancer. The prevalence of GG, GC and CC genotypes of rs799905 between BC cases and controls in Group I (with detected methylation of *BRCA1* in peripheral blood) and Group II (unselected) was similar and was not associated with breast cancer risk (Table 3). However, analysis of clinicopathological features of breast cancer patients from Group II showed a correlation of rs799905 with TNBC and lymph nodes metastases (Table 4). Both observations were of borderline significance (*p* = 0.048 and *p* = 0.046, respectively) and require further investigation. Interestingly, we found a relatively strong and statistically significant association of rs799905 with *BRCA1* germline PV (*p* = 0.003) (Table 4). The prevalence of GG, GC and CC genotypes in carriers of *BRCA1* germline PVs (Group III) differed substantially from Group I and II, showing 10-times lower frequency of CC genotype (Table 3).

Variant rs799905 has been previously studied mostly in the context of allele specific *BRCA1* methylation and used to distinguish methylated and unmethylated alleles in heterozygotes [20–23]. Only one study performed in the Jordanian population analyzed the correlation of rs799905 with prognostic factors and clinical characteristics of breast cancer. However, their study group was relatively small (230 BC patients) and the results, including association of rs799905 with LN metastases and TNBC, were not statistically significant [24].

It has been suggested that rs799905 may be associated with *BRCA1* expression [25]. Variant *BRCA1* c.-20 + 101 C > G is located within a CpG site and the presence of the G allele creates a potential methylation site. Depending on the methylation status of this site, it may influence *BRCA1* gene expression due to its proximity to transcription factor binding sites. Furthermore, it has been demonstrated that specific alleles of rs799905 are consistently linked with a second common variant rs8176071 (TGTT > T) located 818 bp upstream of rs799905. It was shown, that the C allele of rs799905 is associated with the GTT deletion of rs8176071, while the G allele is linked with the presence of GTT. Both variants appear to counterbalance each other, maintaining stable BRCA1 expression. Notably, alternative combinations of these polymorphisms (allele C/TGTT or G/T(delGTT) was reported to induce in cell lines a significant reduction in BRCA1 expression [25]. The rs8176071 was not included in our study, and we could not confirm these observations.

Results of our analyses suggest that variant rs799905 is not directly associated with an increased risk of breast cancer or *BRCA1* promoter methylation, which is generally in line with previously published reports. However, we observed in our study a significant and novel correlation between rs799905 and germline *BRCA1* PVs that remains to be explained. In previous studies it was shown, that methylation of *BRCA1* gene is restricted to non-carriers of *BRCA1* PVs, suggesting that the mutation and methylation of *BRCA1* may be mutually exclusive [26, 27]. It was suggested that the G allele of rs799905 may create a CpG - a potential methylation site. However, we observed in our analysis a higher frequency of the G allele in *BRCA1* carriers (Tables 3 and 4) that appears to contradict this notion, and suggests that this site probably remains unmethylated. Notably, no prior research has focused on the possible association between rs799905 and *BRCA1* PV, and at present, we are unable to explain the underlying mechanism observed in our study between the association of rs799905 and germline *BRCA1* PVs. Further studies, including larger groups of *BRCA1* PV carriers are necessary to investigate and understand the connection between rs799905 and *BRCA1* PVs.

## Conclusions

In this study, we aimed to analyze the *BRCA1* promoter region to identify germline alterations associated with *BRCA1* methylation in peripheral blood DNA. We did not find a genetic variant which could correlate with a *BRCA1* epimutation in the Polish population, and be used as a genetic marker for the identification of a breast cancer predisposition. Sequencing of 5’UTR of *BRCA1* gene in pre-selected breast cancer patients revealed a common variant *BRCA1* c.20 + 101 C > G (rs799905) located within a CpG site, of which the G allele potentially might create a methylation site. Association analyses did not reveal any correlation between rs799905 and *BRCA1* promoter methylation and breast cancer risk. Nevertheless, this variant may be associated with certain clinical features of breast cancer, though further research is required to confirm these findings. In addition, rs799905 may be associated to some extent with *BRCA1* PVs, but the nature and mechanism of this co-occurrence remains unclear. Further research is needed to clarify correlation and its potential biological significance.

## Data Availability

The data that support the findings of this study are available from the corresponding author upon reasonable request.

## Abbreviations

ALFA: Allele frequency aggregator
BC: Breast cancer
DCIS: Ductal carcinoma in situ
IHC: Immunohistochemistry
LN: Lymph nodes
MAF: Minor allele frequency
MS-HRM: Methylation-sensitive high resolution melting
OC: Ovarian cancer
PV: Pathogenic variant
TNBC: Triple-negative breast cancer
UTR: Untranslated region
